# The electro-oxidation of primary alcohols via a coral-shaped cobalt metal–organic framework modified graphite electrode in neutral media

**DOI:** 10.1038/s41598-022-12200-w

**Published:** 2022-05-20

**Authors:** Vahid Khakyzadeh, Salbin Sediqi

**Affiliations:** Department of Chemistry, K. N. Toosi University of Technology, 15875-4416, Tehran, Iran

**Keywords:** Catalysis, Green chemistry, Organic chemistry, Electrocatalysis

## Abstract

The electro-oxidation of alcohols into corresponding aldehydes achieved enormous attention. However, numerous challenges remain in exploring catalytic systems with high conversion efficiency and selectivity. Considering the worldwide attention toward metal–organic frameworks (MOFs) as outstanding crystalline porous materials, many chemists have been encouraged to use them in organic transformations. In this study, a novel coral-shaped cobalt organic framework was grown onto the surface of a functionalized graphite electrode (Co-MOF/C) to fabricate an efficient modified electrode in the electro-oxidation alcohols. The modified Co-MOF/C electrode showed high stability, large surface area, rich pores, and good conductivity as a desirable water-stable working electrode for selective oxidation of alcohols into aldehydes in good to excellent yields under a diffusion-controlled process.

## Introduction

Metal–organic frameworks (MOFs) are a unique class of well-ordered polymeric metal complexes with potential voids that consist of coordination between metal clusters/cations and multidentate organic linkers, arranged in a vast array of geometries affording substantially high porosity and internal surface areas^1^. To date, a wide array of MOFs has been synthesized using different techniques, such as hydrothermal^2^, solvothermal^3^, sono-chemical^4^, microwave-assisted^5^, liquid-phase epitaxy^6^, mechanochemical^7^, and recently, electrochemical approaches^8^. The conventional solvothermal synthesis of MOFs is carried out at atmospheric pressure and lower temperatures compared to the hydrothermal method, allowing diffusion-controlled synthesis. The tunable features of MOFs such as porosity, topology, and functionality^9^, led to their structural and chemical diversity and, consequently, significantly developed their application in a variety of fields, including, drug delivery^10^, purification and separation^11^, gas storage^12^, light-harvesting^13^, energy storage^14^, magnetic materials^15^, chemical sensing^16^, and catalysis^17^. More recently, MOFs have been used for modification of working electrodes to be employed in the voltametric analysis of organic and inorganic species. The higher sorption capability of MOF-modified electrodes could lead to enhanced accumulation of the targeted analyte species, offering several advantages such as high selectivity, exceptionally low detection limits, and simultaneous determination of multiple analytes^18^. However, regardless of many attempts that have been made to use MOFs as an electrode modifier, there is still limited information about the selectivity, conductivity, and stability of MOFs in the working meida^19^ and, therefore, the research for efficient MOF-modified electrodes is still a challenging task.

The chemical functionalization of the electrode surface is essential for producing strong bonds, tailoring the interface properties (proper spacing, orientation, surface density, and stability), and promoting a suitable anchoring of target molecules with diverse nature and structures such as biomacromolecules, polymers, and nanoparticles onto the surface^20^. Thus, reasonable control at this initial level plays a key role in the performance of the final modified electrodes. Among various surface functionalization approaches, the reduction of aryldiazonium salts has been considered a straightforward, rapid, and versatile methodology that allows a strong covalent attachment of various chemical electroactive functions onto a conductive substrate by changing the substituents of the aryl ring^21^. Also, the resulting functionalized electrodes have shown resistance to heat, ultrasonication, and chemical degradation^22^. Many different strategies have been employed for grafting aryl derivatives^23^, including electrochemistry, photochemistry, microwave, ultrasonication, and reduction by chemical agents, while the electrochemical method has become the preferred choice since the deposition process could be readily controlled and adapted to the substrate^24^. In general, electro-reduction of aryldiazonium salts involves the concerted formation of aryl-based radicals at the vicinity of the electrode and the elimination of dinitrogen upon reduction. Subsequently, the highly reactive aryl radicals can either make a covalent binding to the electrode surface or to already grafted moieties. As a consequence, electro grafting of aryl moieties can be directed toward the synthesis of well-ordered monolayers or disordered multilayers by adjusting the experimental conditions^25^.

**Figure 1:**
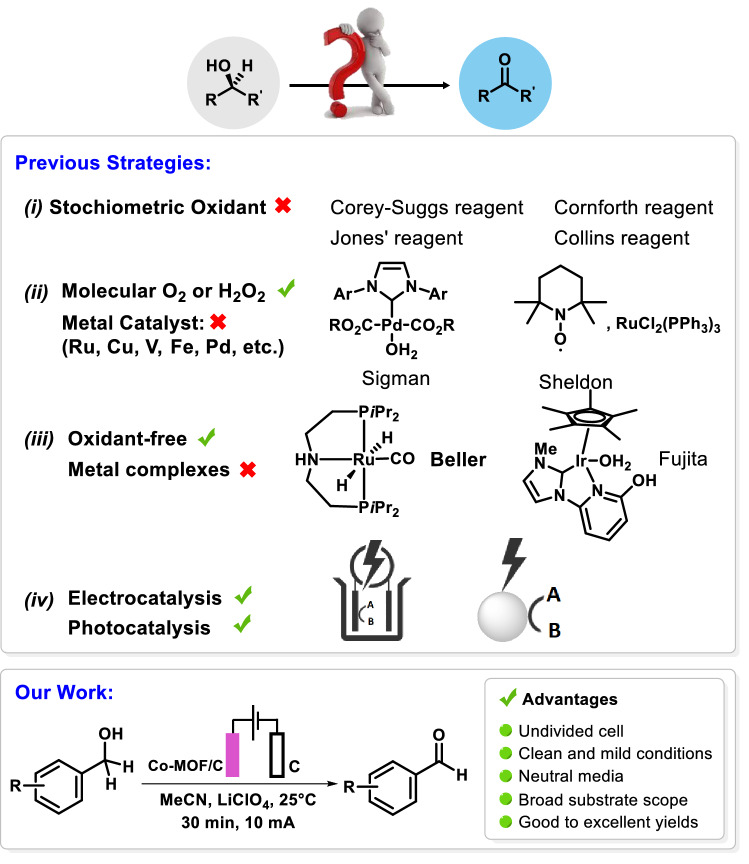
Previous strategies and our work for alcohol oxidation.

Aldehydes are not only fundamental building blocks of numerous biologically active compounds but also paramount reagents in modern organic syntheses^26^. So far, various methods have been employed for the oxidation of alcohol to aldehydes as follows (Fig. 1): (i) the use of stoichiometric oxidants (chromium-based oxidant, Dess-Martin Periodinane (DMP), Manganese oxides, Swern oxidizing agents, etc.)^27^; (ii) Molecular oxygen, hydrogen peroxide (H_2_O_2_), or air in combination with proper transition metal catalysts (Ru, Cu, Ir Au, V, Fe, or Pd)^28^; (iii) oxidant-free alcohol oxidation with hydrogen evolution using transition metal catalysts and sophisticated ligands^29–34^; (iv) alcohol oxidation via photo-catalysis^35^ or electrocatalysis^36–41^.

Despite the efficiency of the first three approaches, they usually suffer from serious disadvantages, including metal waste production, harsh reaction conditions, undesirable side products, moisture-sensitive reagents, stoichiometric amounts of oxidants, and expensive materials while the last method, photo-catalysis or electrocatalysis, has the merit of performing reactions under green and mild conditions. According to the perspective of pharmaceutical manufacturers, developing a green approach toward the oxidation of alcohols holds 4^th^ rank in the top chemistry research areas and, therefore, introducing green, cost-effective and productive oxidation methods are still highly demanded for pharmaceutical and chemical industries^42^. Recently, few attempts have been made towards the preparation of modified electrodes in order to use them in alcohol electrooxidation reactions^43–45^. Herein, an unprecedented MOF-modified graphite electrode was designed and fabricated through two step modification process involving the electro grafting of 4-carboxyphenyl, via a process of electro-reductive dediazoniation of aryldiazonium salt, followed by the assembling of a Cobalt-based MOF onto the surface of 4-carboxyphenyl functionalized graphite. Then, the performance of the modified electrode was investigated in the direct electro-oxidation of alcohols to aldehydes, as working electrode.

## Results and discussion

### Fabrication of Co-MOF modified glassy carbon electrode (GCE)

**Scheme 1:**
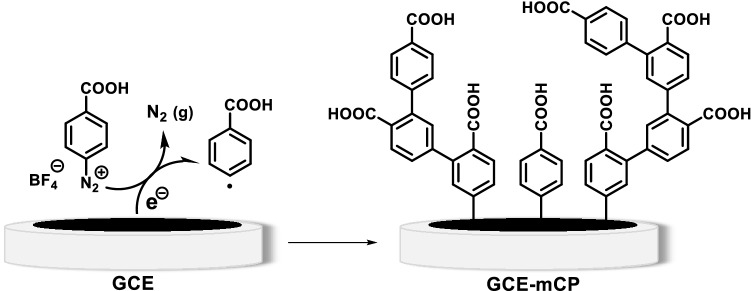
Electrografting of 4-carboxyphenyl diazonium salt onto the GCE surface.

The fabrication process of the Co-MOF modified GCE (Co-MOF/GC) electrode comprises two consecutive steps. At first, the surface of the GCE was chemically functionalized with 4-carboxyaryl moieties through the electro-reductive dediazoniation of 4-carboxyphenyl diazonium salt (4-CPD) (Scheme 1).

**Figure 2:**
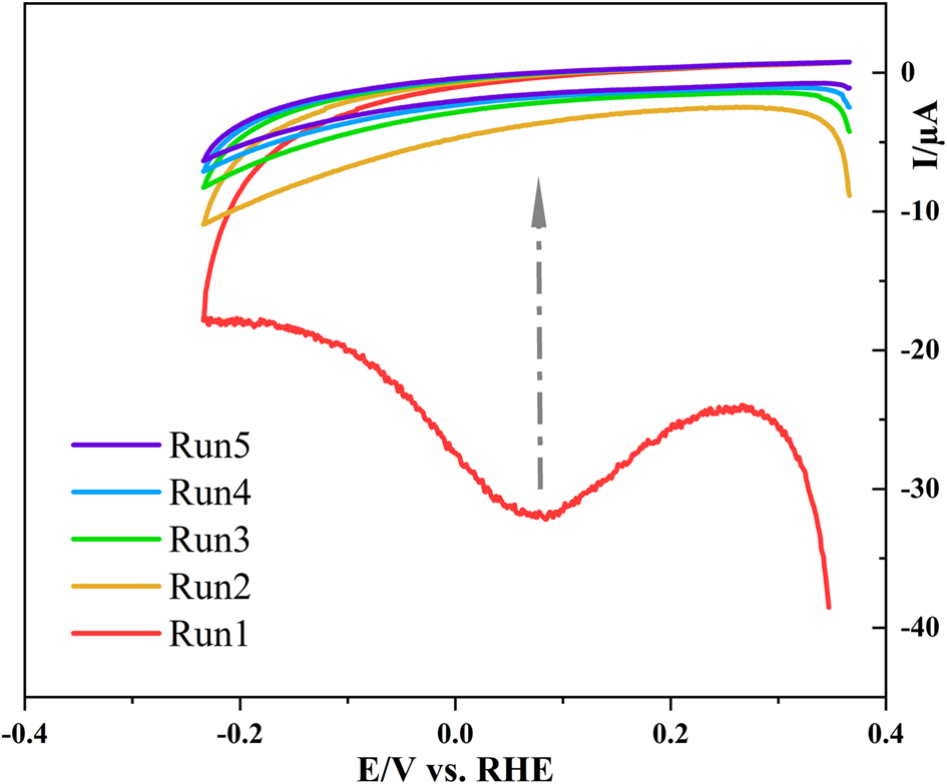
Multi-cyclic voltammogram of 0.4 M 4-carboxyphenyl diazonium salt at the surface of GCE in acetonitrile solution containing 0.1 M LiClO4, scan rate: 100 mV/s.

In the next step, the 4-carboxyphenyl functionalized electrode, namely (GCE-mCP), was immersed in the dimethylformamide (DMF) solution containing trimesic acid (TMA) and Co(NO_3_)_2_ and transferred to an autoclave at 120 °C to fabricate the desired Co-MOF/GC electrode. In this regard, cyclic voltammograms (CVs) were recorded for surface functionalization of GCE by electrochemical reduction of 4-carboxyphenyl diazonium salts (0.4 mM) in acetonitrile solution containing lithium perchlorate (0.1 M LiClO_4_) by applying the optimum potential range of + 0.35 V to −0.25 V (vs RHE) at the scan rate of 100 mV/s. Figure 2 shows five consecutive CVs of GCE during electrochemical functionalization. The reductive C_0_ peak observed in the 0.08 V positive going scan arises from the electro-reduction of a diazonium salt generating the aryl radicals through the elimination of N_2_ gas.

The aryl radicals subsequently link to the GCE surface via covalent bonding to result in a highly stable functionalized layer on the surface of the electrode. The adequate concentration of 4-CPD and moderately low scan rates led to a continuous reduction of the 4-CPD, affording the formation of carboxyphenyl multilayers at the surface of the GCE^46^. The curve was irreversible due to the loss of N_2_ and it is broad because the surface was being modified during the voltammogram. The insignificant peak current of the cathodic curve in subsequent potential scans was attributed to the further adsorption of 4-carboxy phenyl moieties and formation of insulating organic film on the electrode which could cause the electrode surface blocking^47^.

After the electro grafting of 4-carboxyphenyl on the surface of GCE, the organic functionalized electrode was immersed in the DMF solution containing Co(NO_3_)_2_. 6 H_2_O and Trimesic acid and transferred to autoclave for 16 h at 120 °C to fabricate the desired Co-MOF/GC electrode.

### Electroactivity of Co-MOF modified GCE

**Figure 3:**
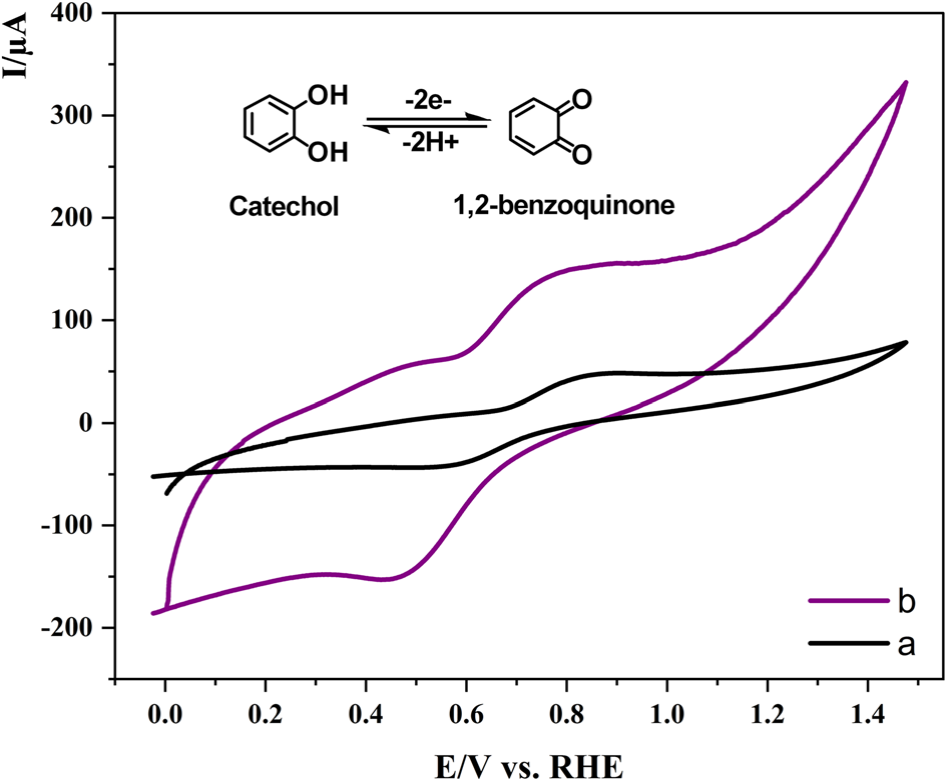
Cyclic voltammograms of 1 mM catechol at the surface of (a) GCE and (b) Co-MOF/GCE in aqueous phosphate buffer solution (pH = 3), scan rate: 100 mV/s.

In order to investigate the success in the electrode modification process, as well as the initial evaluation of electrochemical behavior of the Co-MOF/GCE toward alcohol oxidation, some voltammetric studies were done (Fig. 3). In this regard, the voltammetric response of catechol oxidation as a typical electroactive species was examined on the Co-MOF/GCE modified electrode (curve b) and compared to the nonmodified bare GCE response (curve a). As is evident, the Co-MOF/GCE showed a considerable redox current compared to the bare GCE electrode, implying the higher conductivity of the modified electrode, as well as the higher accessible surface area of the MOF structure, which are two decisive factors in the electrocatalytic performance of the modified electrode. A couple of peaks observed in the voltammograms arise from oxidation of catechol to 1,2-Benzoquinone and vice versa within the 2e^−^/2H^+^ process.

**Figure 4:**
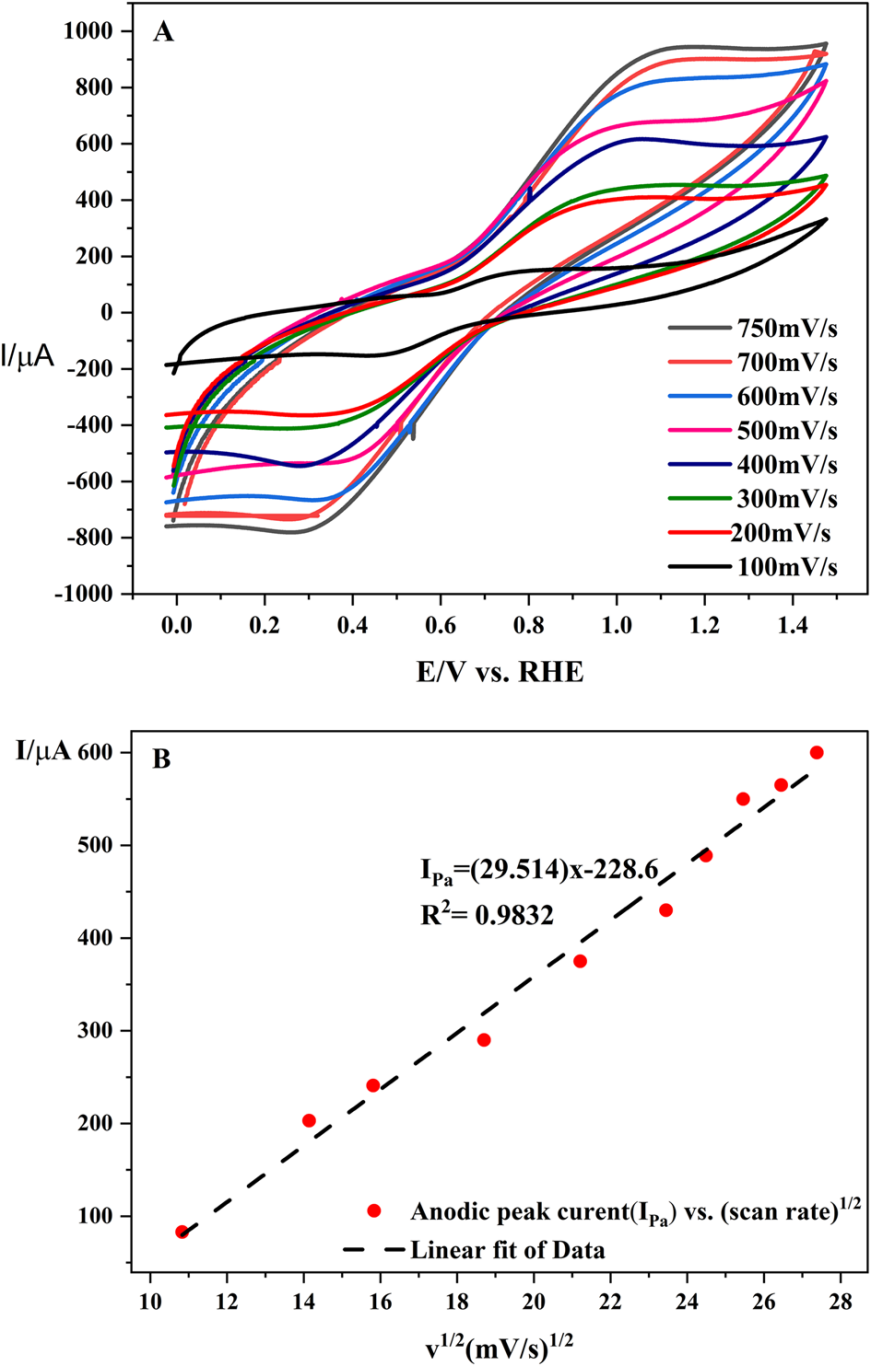
(**A**) CVs of 1 mM catechol obtained for the different scan rate analysis (100–750 mV/s) at the surface of Co-MOF/GCE in aqueous phosphate buffer solution (pH = 3); (**B**) The calibration plot of cathodic peak current versus square root of scan rates.

Next, the cyclic voltammograms of Co-MOF/GCE at various scan rates were recorded to further clarify electrochemical performance of the electrode (Fig. 4A). A slight increase of peak-to-peak separation by augmentation of scan rate proves facilitation of electron and ion transfers in Co-MOF/GCE even at higher scan rates. The peak current values were plotted against the square root of the scan rate (υ^1/2^) values in Fig. 4B. The linear relationship between the peak current values and the square root of the scan rate obtained from the linear regression equations of $Ipa = 29.514 x {-} 228.6$ with the correlation coefficient values of R^2^ = 0.9832 indicates that the oxidation of catechol at the surface of Co-MOF/GCE is a diffusion-controlled process and the Randles−Sevcik equation is applicable.

**Figure 5:**
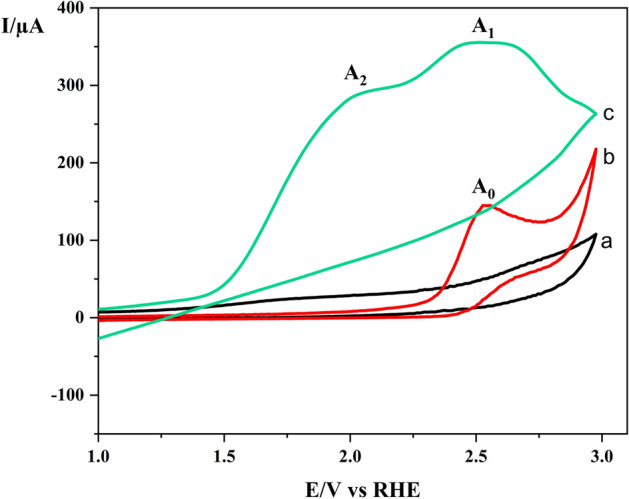
Cyclic voltammogram of GCE in acetonitrile solution containing (0.1 M LiClO4) (a) in the absence of BA; (b) in the presence of 10 mM BA; (**c**) CV of BA at Co-MOF/GCE in the same conditions of b, scan rate: 100 mV/s.

Moreover, the electrocatalytic behavior of Co-MOF modified GCE was examined toward the oxidation of benzyl alcohol (BA). Figure 5 (curve a) shows the CV of unmodified GCE in a blank acetonitrile solution containing only supporting electrolyte (0.1 M LiClO_4_) wherein no anodic and cathodic peaks were obtained. Also, the cyclic voltammogram was recorded using unmodified GCE in the presence of 1 mM BA at the same condition of curve a (Fig. 5, curves b). The anodic peak (A_0_) observed in curve b with an irreversible feature at 2.54 V (vs. RHE) arises as a result of BA oxidation to the corresponding benzoic acid. Finally, the cyclic voltammogram was recorded for the oxidation of BA using the Co-MOF modified electrode at the same reaction condition of curve b, wherein some noticeable differences were evident as follows. (i) the height of anodic peaks current (Fig. 5, curve c) increases several folds compared to curve b, indicating the extensive and available surface area for electroactive material; (ii) the voltammogram demonstrates two successive electron transfers (peak A_1_, 2.08 V) and (peak A_2_, 2.52 V), which are related to the oxidation of BA to benzaldehyde and benzaldehyde to benzoic acid, respectively. The obtained results imply that the oxidation of BA is more controllable while using the Co-MOF modified GCE compared to bare GCE and, therefore, by tuning the reaction condition the oxidation process could be driven toward the synthesis of benzaldehyde rather than benzoic acid.

### Fabrication of Co-MOF modified graphite electrode

**Figure 6:**
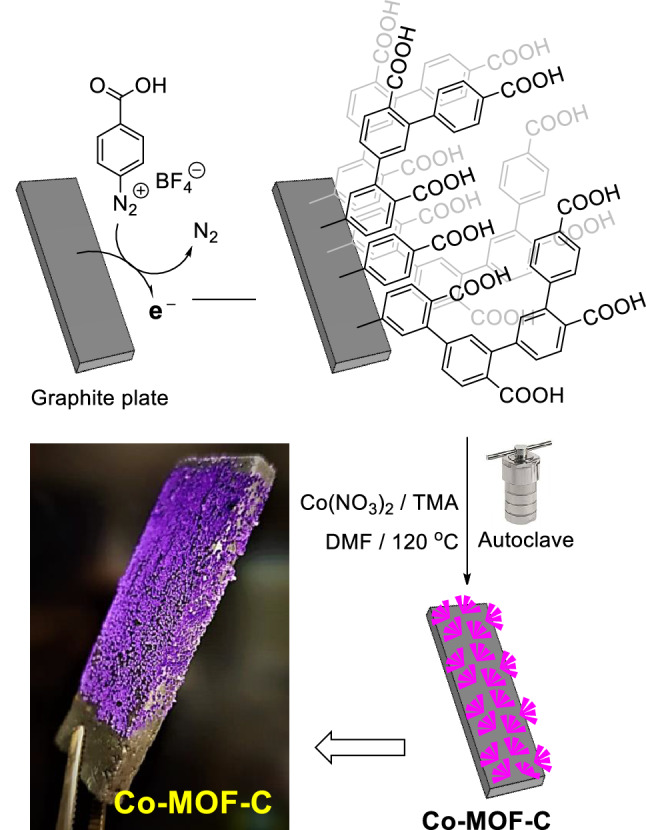
Schematic illustration of graphite electrode modification process.

Considering the data obtained from voltammetric studies, in the next step, it has been strived to prove that the presented procedure is applicable for the large-scale fabrication of Co-MOF modified graphite electrode (Co-MOF-C). In this regard, the graphite electrode modification process involving 4-carboxyphenyl functionalization of GCE surface through electroreduction of corresponding diazonium salt followed by preparation of Co-MOF crystalline onto the functionalized GCE by transferring it to an autoclave containing Cobalt source and Trimesic acid in DMF at 120 °C for 16 h, was conducted to fabricate the desired Co-MOF-C electrode, which appeared in the form of violet-colored coral-shaped crystals onto the surface of graphite electrode. The schematic illustration of the presented electrode modification process is shown in Fig. 6.

### Structure characterization of the Co-MOF-C

The physicochemical properties of the as-prepared Co-MOF-C electrode, as well as its structure, was fully characterized using the following analyses: The differential scanning calorimetry (DSC), X-ray powder diffraction (XRD), Field emission scanning electron microscopy-energy dispersive X-ray mapping (FESEM-EDX mapping), Fourier transform infrared (FT-IR), and N_2_ adsorption–desorption isotherm measurements.

### XRD characterization

The XRD pattern of Cobalt-MOF modified graphite electrode {Co-MOF-C} exhibited a pattern due to its crystal structure at 2θ = 10.2, 13.6, 16.1, 19.3, 23.6, 25.3, 29.8, and 55.2◦. Peaks with orient along the (111), (002), and (002), directions related to Cobalt crystals which are in agreement with the values reported by the JCPDS 110,692 card. Also, peaks at 2θ = 25.3, and 55.2◦ with orient along the (002), and (004) directions, respectively are referred to the graphite (S1 in supplementary information)^48^.

### FT‑IR measurement

The FTIR spectra was carried out to detect the functional groups and characterize covalent bonding information of the Co-MOF (S2 in supplementary information). The spectrum of Co-MOF shows a broad peak in 3400–3500 cm^-1^ is related to the stretching vibration of hydroxyl groups^49^. The Peaks in the range of 1450–1650 cm^-1^ and 650–900 cm^-1^ are related to the stretching and bending vibrations of C = C in aromatic Trimesic acid linker. It is evident the peak at 1721 cm^-1^ related to stretching vibration of C = O in Trimesic acid has shifted to 1622 cm^-1^ in the Co-MOF spectrum due to the coordination of oxygen to cobalt (Red spot to purple spot), which weakens the strength of C = O bond in the carbonyl^50^. Also, the peaks observed in the range of 1250–1500 cm^-1^ in the Co-MOF spectrum are closer to each other compared to the peaks obtained in trimesic acid spectrum indicating that the structure has become more rigid and organized^51^. Moreover, we found that the peak at 1250 cm^-1^ in trimesic acid disappeared in Co-MOF spectrum (blue spot).

### DSC analysis

The DSC measurement of the Co-MOF-C indicates that the first step of weight loss starts from 111.7 to 150 °C, which can be attributed to the evaporation of gaseous molecules such as water absorbed by porous structure from the air. Subsequently, another peak from 150 to 195 °C shows the evaporation of DMF from the MOF pores. Finally, the endothermic peak observed at 247 °C suggests that the structure of MOF crystals turns amorphous at this point indicating the thermal stability of MOF is up to 247 °C (S3 in supplementary information).

### FESEM images and EDX mapping analyses

**Figure 7:**
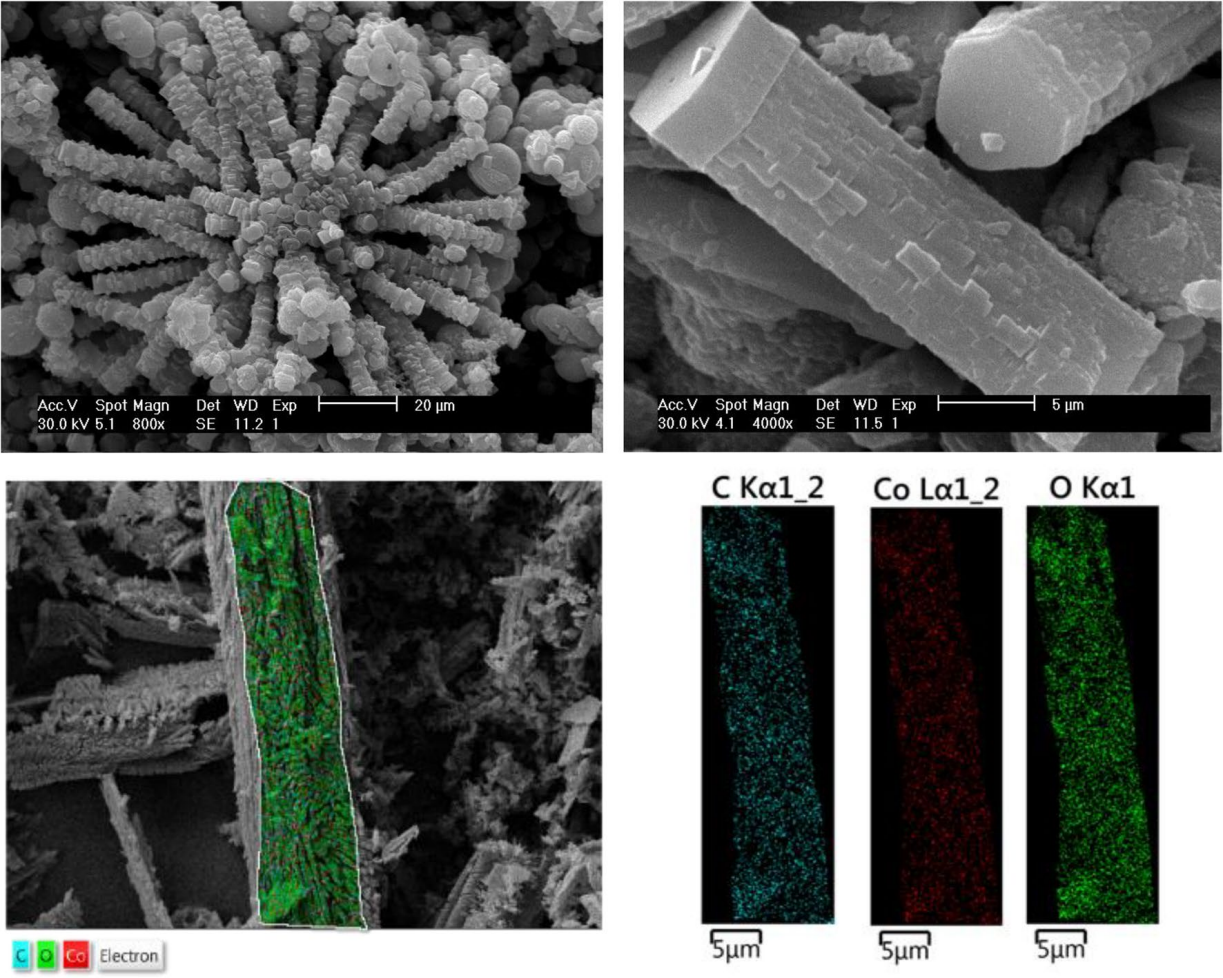
FESEM images and EDX mapping analyses of Co-MOF-C.

The morphological characteristics of the Co-MOF-C were represented using FESEM analysis (Fig. 7, top). In the high-resolution FESEM images, it can be clearly observed that the fabricated framework possesses rod-shaped crystals that are set next to each other in structures resembling coral reefs. It was also revealed that these rod-shaped crystals with the smallest width of approximately 7 mm, the largest width of 8.24 mm, and a length of 26.8 have a hexagonal cross-section. The EDX spectra and the elemental mapping of Co-MOF are displayed in (Fig. 7, down). The images reveal a homogeneous distribution of elements (Co, O, and C) corresponding to Co-MOF, which were also depicted in EDX spectra, implying that MOF crystals are uniformly distributed on the surface of the graphite electrode.

### The pH stability

Given that the stability of MOFs in the working medium plays a significant role in its applicability as an electrode modifier, it is important to investigate the stability of presented MOF in acidic, neutral, and basic media. The MOF degradation in water occurs due to a series of substitution reactions, in which water or hydroxide ions replace the metal-coordinated linkers. In Comparison to neutral water molecules, proton (H^+^) and hydroxide (OH^−^) ions are far more destructive to MOFs. In acidic media, the decomposition of MOFs mainly originates from the competition of H^+^ and metal ions for the coordinating linkers, whereas in basic media, the OH^−^ competitively binds to the metal cations of MOFs and replaces organic linkers, causing MOF degradation^52^. In this regard, the stability of Co-MOF was investigated in different pHs using hydrochloric acid and trimethylamine to adjust the pH values. The results showed that the as prepared Co-MOF was unstable in pHs below 1, however, it proved to be stable in pH values between 1 to 10. Also, some color changes were observed in pH ranges above 10, implying that the presented Co-MOF is stable in a wide range of pHs that is also another important factor in the applicability of Co-MOF-modified electrodes in various mediums (S4 in supplementary information)^53^.

### N_2_ adsorption–desorption isotherms

**Figure 8:**
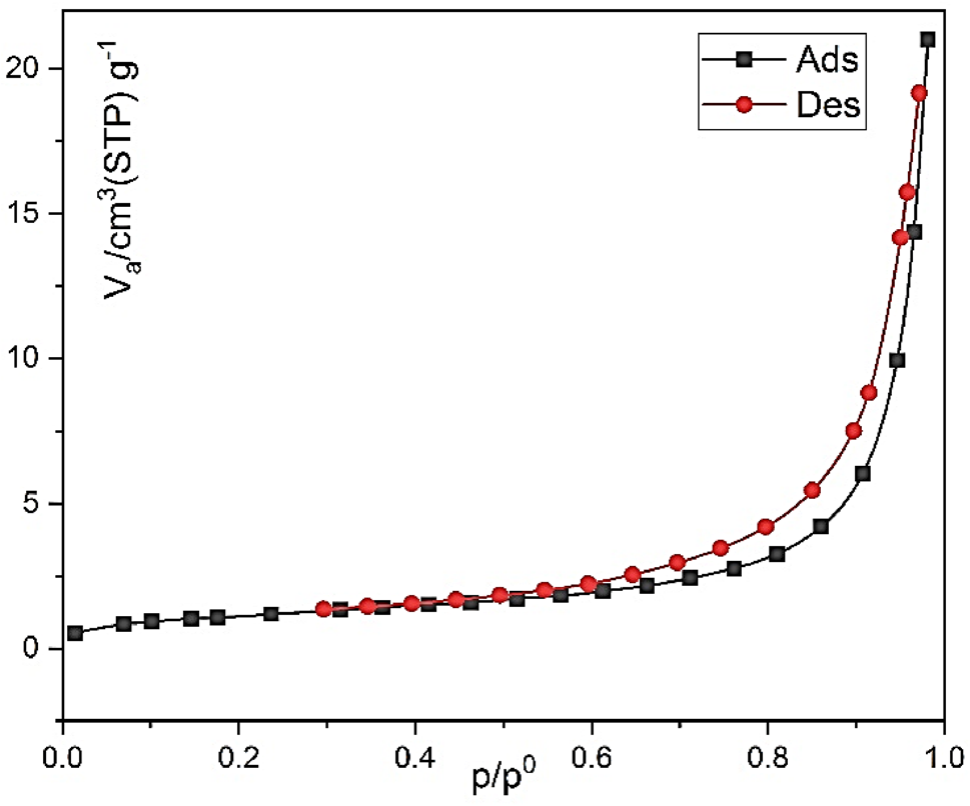
BET analysis of Co-MOF-C (N2 adsorption–desorption isotherm curve).

Surface area analysis of Co-MOF-C was conducted by nitrogen adsorption–desorption isotherms at 77 K in order to assess their porosities and surface areas. The total catalyst surface area was 1200 m^2^/g. As it is shown in Fig. 8, according to the IUPAC classification, the sample exhibited typical **IV** type isotherms at the borderline with type **II** and **H3** type hysteresis loops at high relative pressures. This type of isotherm suggests the presence of mesopores with a pore size distribution continuing into the macropore domain. Also, the type **H3** hysteresis is usually observed on solids inclusive of some particle agglomeration which leads to slit-shaped pores with 3.35 nm average pore diameter.

### Application of Co-MOF modified graphite electrode in electro-oxidation of alcohols

**Scheme 2:**
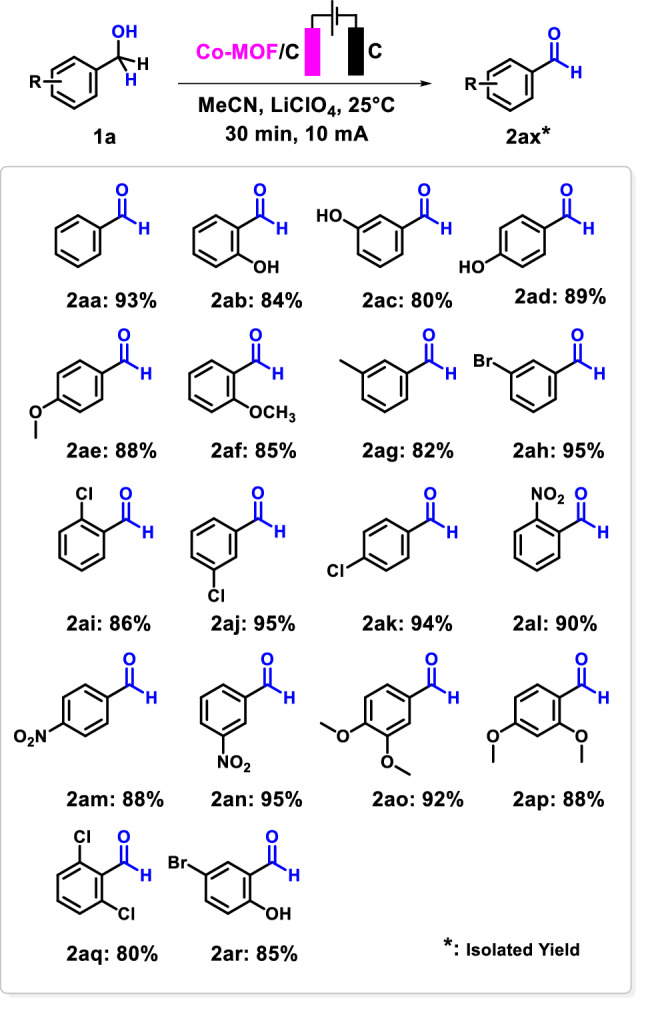
Reaction scope of the direct electro-oxidation of alcohols to the corresponding aldehydes using Co-MOF-C electrode in neutral media.

In order to investigate the electrocatalytic performance of the Co-Modified graphite electrode toward the selective oxidation of benzyl alcohol derivatives, the electrooxidation reaction was performed at room temperature via a two-electrode undivided cell system (Co-MOF modified graphite anode and graphite cathode) containing alcohol (1 mmol), acetonitrile, and LiClO_4_ electrolyte (0.1 M), under current-controlled coulometry condition (10 mA). As is evident in the table of products, the reactions have been catalyzed efficiently and the aldehydes are produced in high yields (80–95%) within a short reaction time (around 30 min). As it is clear from the product scope, the results showed that better yields were obtained when benzene rings, in benzyl alcohol, have electron-withdrawing groups such as halogens (–Br and –Cl), and –NO_2_ and benzyl alcohol with electron-donating groups gave lower yields. Moreover, benzyl alcohols including two groups were tolerable in the introduced reaction condition (Scheme 2).

## Conclusions

In this work, efficient electrochemical oxidation of benzyl alcohol derivatives to desired benzaldehydes, in the neutral media, was developed by a new modified graphite electrode (Co-MOF-C). Graphite was successfully modified by a 2-step procedure involving the electro grafting of 4-carboxyphenyl via electro-reductive dediazoniation of aryldiazonium salt followed by a solvothermal synthesis of a coral-shaped violet-colored Cobalt metal–organic framework onto its surface. High stability, conductivity, and also a high assessable surface area were some impressive features of the modified electrode in the diffusion-controlled electro-oxidation of benzyl alcohols.

## Methods

All used materials including, acetonitrile, Lithium perchlorate, 4-Amino Benzoic acid, Sodium nitrite, Methanol, 1,3,5-benzene tricarboxylic acid, and alcohols were purchased from Merck. Hydrochloric acid, Potassium tetrafluoroborate, Co(NO_3_)_2_. 6H_2_O, Dimethylformamide were purchased from Sigma-Aldrich. All chemicals have been used without further purification.

### Synthesis of 4-carboxyphenyl diazonium salt

The preparation procedure started by dissolving 4 mmol Potassium Fluoroborate (KBF_4_) in 5 ml methanol and stirring it for 30 min. Next, 4 mmol HCl, 37%, was added to the mixture and followed by another 30 min of stirring, which resulted in Potassium chloride (KCl) precipitation in the Fluoroboric acid (HBF_4_)-containing solution. The HBF_4_ was separated by simple filtration using filter paper. Afterward, 2 mmol of 4-amino benzoic acid was dissolved in HBF_4_ solution and stirred while being in an ice bath for 30 min. Then sodium nitrite (NaNO_2_ 2 mmol) dissolved in 5 ml water was added to the solution and kept cold at 0 °C for the next steps of the study.

### Modification of glassy carbon electrode

Prior to the electrode modification process, the surface of the GCE was polished by alumina powder slurry on a cotton textile. After each polishing, the electrode was washed with ultrapure water and sonicated in acetonitrile for 1 min for further cleaning and eliminating of all species that might be adsorbed on the GCE surface. Before electrodeposition, the GCE was once treated with acetonitrile. 4-carboxyphenyl functionalization process of the GCE was carried out by electro-reductive dediazoniation of 4-carboxyphenyl diazonium salt (0.4 mM) in deaerated acetonitrile containing LiClO_4_ (0.1 M) as electrolyte. The GCE, Platinum wire and Ag/AgCl electrodes were used as working, counter and reference electrodes, respectively. Cyclic voltammograms were recorded in the potential range from + 0.35 V to − 0.25 V (vs. RHE). After electrochemical functionalization, the modified GCE was washed with acetonitrile and dried, then added to a DMF solution (25 ml) containing Co (NO_3_)_2_.6 H_2_O (0.12 mM) and Trimesic acid (0.12 mM) and put in autoclave for 16 h in 120 °C to give the desired Co-MOF modified GCE.

### Modification of graphite electrode

Prior to modification of the graphite electrode, the graphite was treated with fine-grit sandpaper to remove any possible contaminations on its surface. In the first step of modification, by applying the optimum constant cathodic potential of − 0.45 V (vs RHE) to the graphite electrode for 30 min in an acetonitrile solution containing 4-carboxyphenyl diazonium salt (0.4 mM) and LiClO_4_ (0.1 M) as electrolyte, the electrochemical reduction of mentioned salt led to the generation of carboxyphenyl radicals and its covalent linkage at the surface of the graphite electrode. The steel wire was used as a counter electrode in this step. After electrochemical functionalization, the graphite was washed with acetonitrile and dried. In the second step the 4-carboxyphenyl functionalized graphite was immersed in the DMF solution containing Co(NO_3_)_2_. 6 H_2_O (0.12 mM) and trimesic acid (0.12 mM) and put in autoclave for 16 h in 120 °C to give the desired Co-MOF modified graphite electrode with the mass loading of 1.2 mg/cm^2^.

### General procedure of Aldehyde preparation

The oxidation of alcohols (1 mmol) was carried out in 5 mL of acetonitrile containing LiClO_4_ as electrolyte (0.1 M) via a two-electrode undivided cell system involving Co-MOF modified graphite anode and graphite plate cathode, under controlled-current coulometry. The constant current of 10 mA was applied for 30 min at room temperature. In order to obtain the desired pure products, subsequent to acetonitrile evaporation via rotary evaporator, the precipitated residual was dissolved in mixed solution containing water and ethyl acetate with ratio of (2:8) and the corresponding aldehydes separated from electrolyte through a simple decantation process and purifying compounds were done by column chromatography.

## Supplementary Information

Supplementary Information.

## Data Availability

All data generated or analysed during this study are included in this published article and its supplementary information file.
