# Supplementary material for: The electro-oxidation of primary alcohols via a coral-shaped cobalt metal–organic framework modified graphite electrode in neutral media
Source: Sci Rep. 2022 May 20;12:8560. doi: 10.1038/s41598-022-12200-w (PMC9122998; doi:10.1038/s41598-022-12200-w)
Supplement: Supplementary file 1 — Supplementary Information. [file 41598_2022_12200_MOESM1_ESM.docx]

The Electro-oxidation of Primary Alcohols via a Coral-Shaped Cobalt Metal-Organic Framework Modified Graphite Electrode in Neutral Media

Vahid Khakyzadeh,*^a^ Salbin Sediqi ^a^

*^a^* Department of Chemistry, K. N. Toosi University of Technology, Tehran 15875-4416, Iran;

***Corresponding author email: [v.khakyzadeh@kntu.ac.ir](mailto:v.khakyzadeh@kntu.ac.ir)

| **S0** | Instrumental | 1 |
| --- | --- | --- |
| **S1** | The XRD pattern of Co-MOF-C electrode | 2 |
| **S2** | The FTIR of Co-MOF-C electrode | 2 |
| **S3** | The DSC analysis of the Co-MOF-C electrode | 3 |
| **S4** | The pH stability of the Co-MOF-C electrode | 3 |
| **S5** | The EDX spectra of the Co-MOF-C electrode | 4 |
| **S6** | The picture of Co-MOF-C electrode | 4 |
| **S7** | ^13^C & ^1^H NMR Spectrums | 6 |

**S0.** Instrumental:

Field emission scanning electron microscopy, Energy-dispersive X-ray spectroscopy, and elemental mapping analysis were conducted by a (FESEM-MAPPING, Oxford Instruments, England). X-ray powder diffraction (XRD) patterns were recorded on a Philips PW 1730 instrument to examine the crystal phase of the samples. The FTIR spectra were recorded by a Bruker Tensor 27 FTIR spectrometer using KBr pellets for sample preparation. The surface area analysis was measured by the Nitrogen adsorption-desorption technique using a PHSCHINA device (PHS-1020, China). Voltametric studies were carried out in a classical three-electrode undivided cell system including a platinum wire auxiliary electrode, the Co-modified/unmodified GC working electrode with a geometric surface area of 3.14 mm^2^, and a double junction Ag/AgCl reference electrode. Electrosynthesis of aldehyde derivatives was performed in an undivided homemade two-electrode cell in which the Co-Modified graphite and graphite plate were the working and auxiliary electrodes, respectively. For the preparative electrolysis reactions, the BEHPAJOOH (Isfahan, Iran) coulometer with a maximum current of 300 mA was utilized. All of the electrodes used in this study were manufactured by AZAR Electrode Company, Urmia, Iran.

**S1.** The XRD pattern of Co-MOF-C electrode.


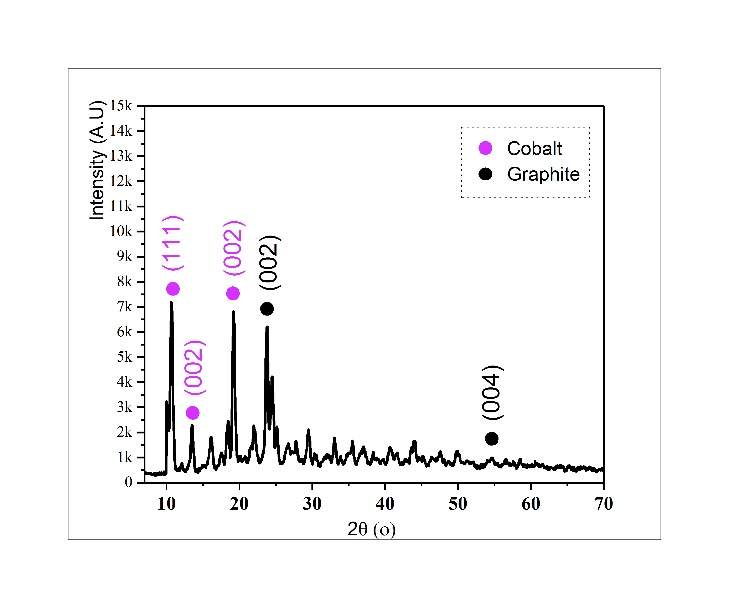


**S2.** The FTIR of Co-MOF-C (purple curve) and Trimesic acid (black curve).


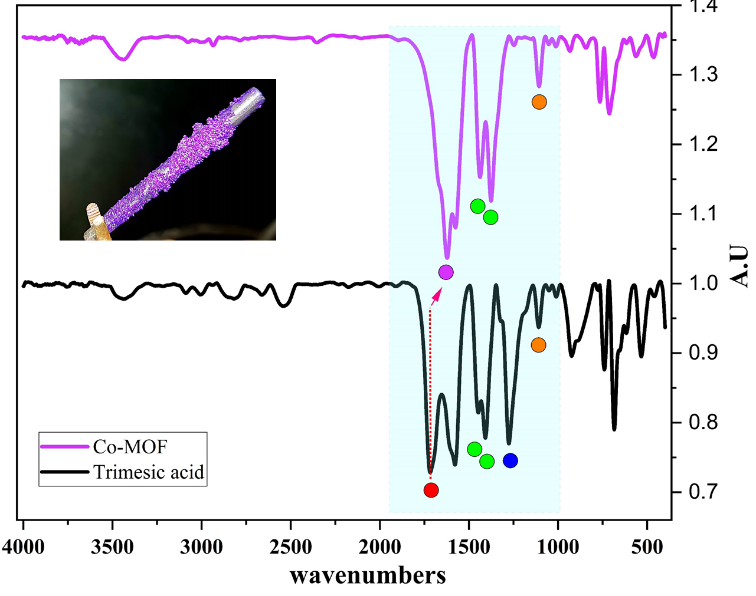


**S3.** The DSC analysis of the Co-MOF-C electrode.

**
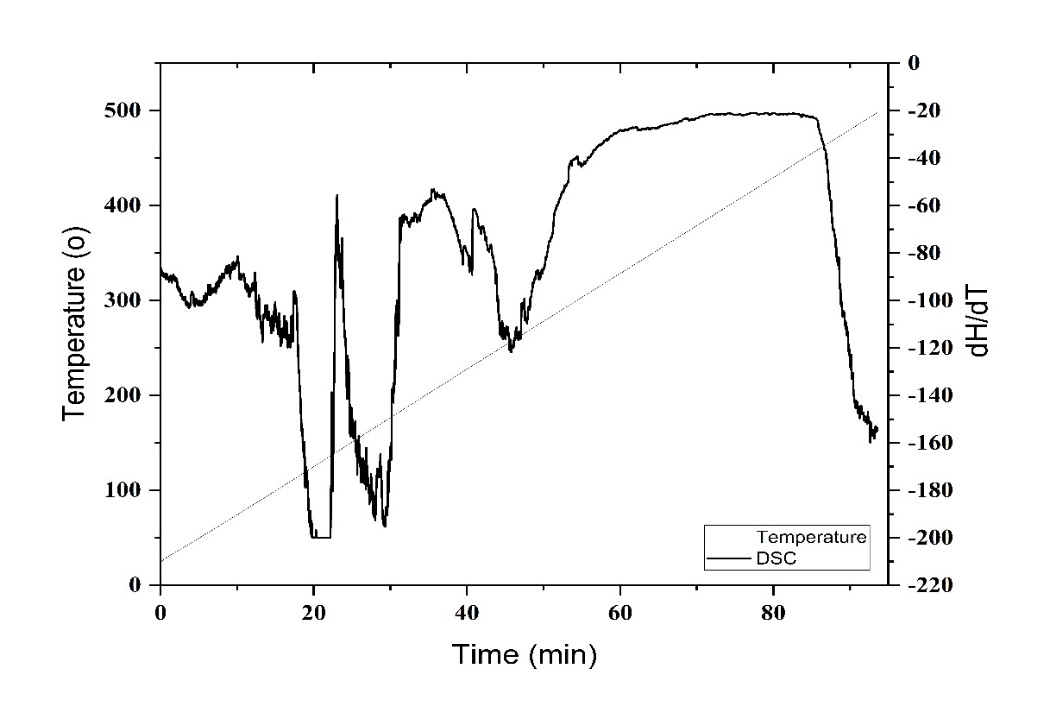
**

**S4.** The pH stability of the Co-MOF-C electrode.


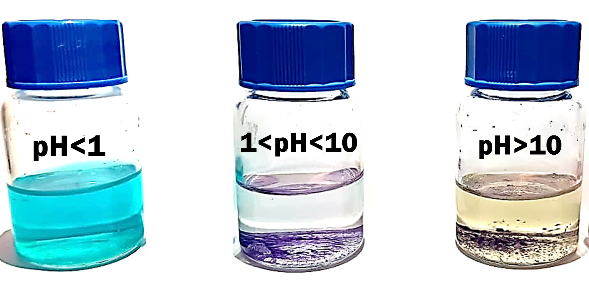


**S5.** The EDX spectra of the Co-MOF-C electrode.


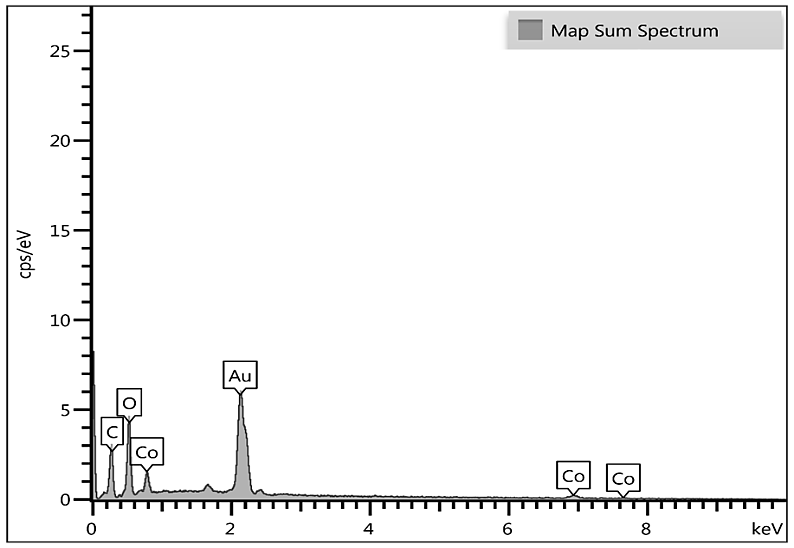


**S6.** The picture of Co-MOF-C electrode after washing with Acetone.


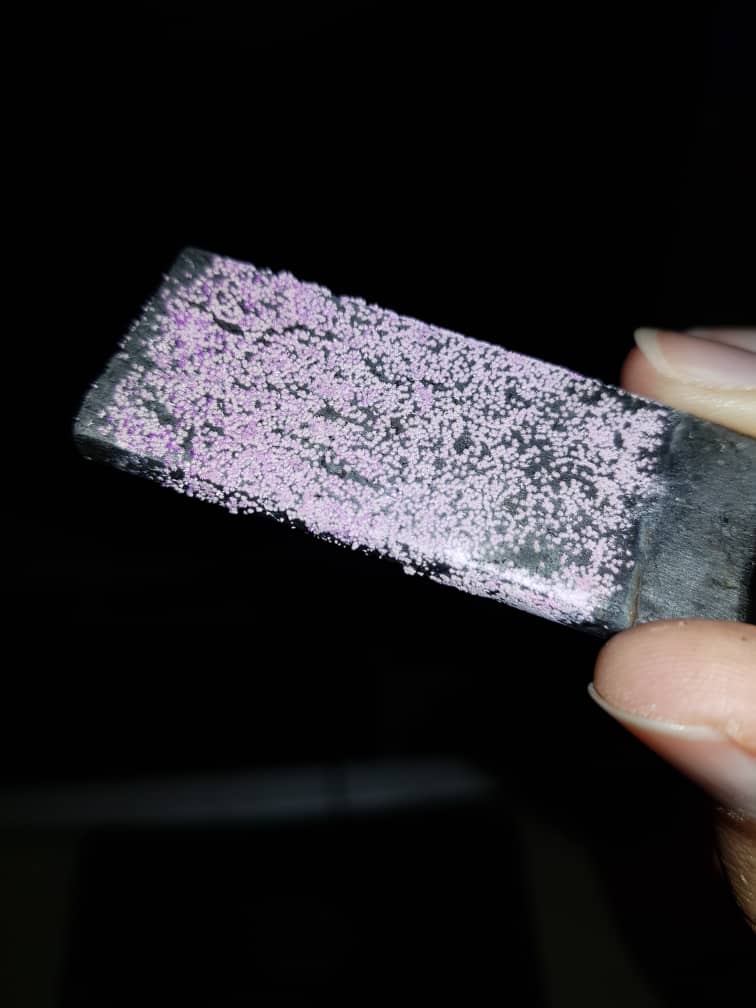


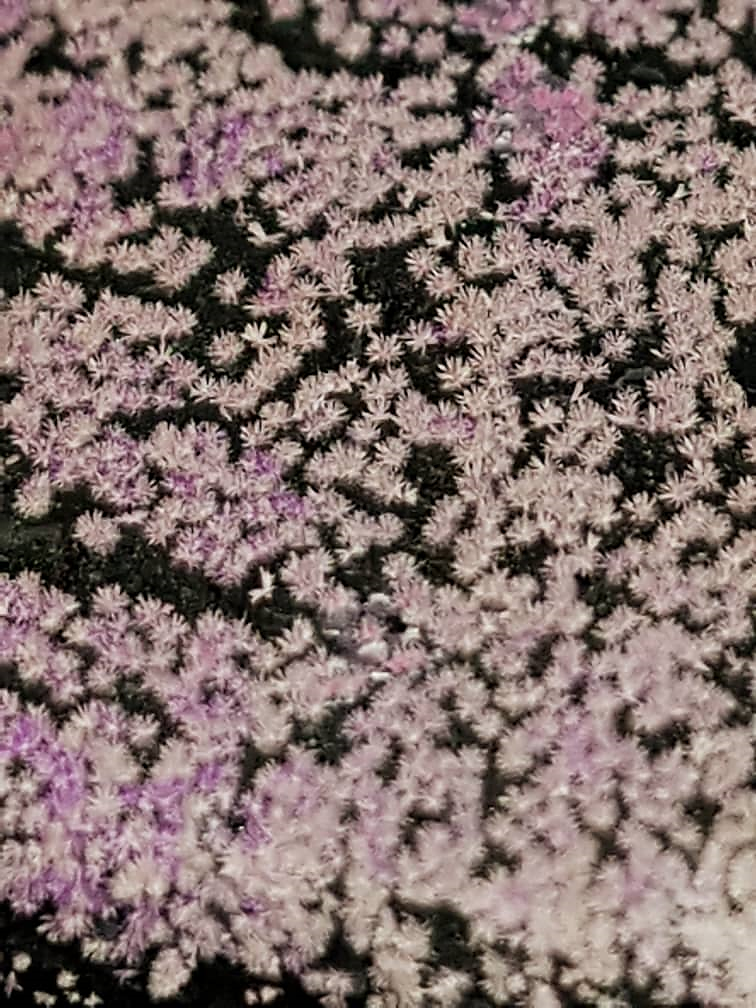


**S7.** ^13^C & ^1^H NMR Spectrums.

**2-Hydroxy benzaldehyde**

**4-methoxy benzaldehyde**

**3-methyl benzaldehyde**

**3-chloro benzaldehyde**

**benzaldehyde**

**3-Hydroxybenzaldehyde**

**3-Bromobenzaldehyde**

**2-Chlorobenzaldehyde**

**2,6-dichloro benzaldehyde**

**5-Bromo 2-Hydroxy benzaldehyde**


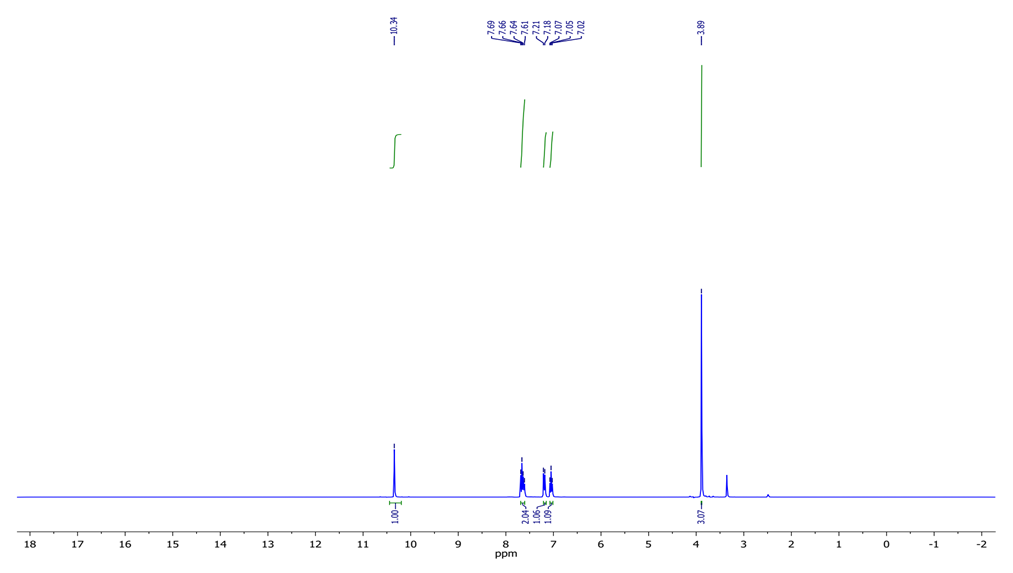

**2-Methoxy benzaldehyde**

**2-Nitrobenzaldehyde**

**4-Nitro benzaldehyde**

**3,4-diMethoxy benzaldehyde**

**3-Nitrobenzaldehyde**

**2,4-dimethoxy Benzaldehyde**

**4-chloro Benzaldehyde**

**4-Hydroxy Benzaldehyde**
